# *N*-(3-Chloro-4-hy­droxy­phen­yl)acetamide

**DOI:** 10.1107/S2414314625006959

**Published:** 2025-08-05

**Authors:** Rao M. Uppu, Frank R. Fronczek

**Affiliations:** a Department of Environmental Toxicology Southern University and A&M College Baton Rouge Louisiana 70813 USA; b Department of Chemistry Louisiana State University,Baton Rouge Louisiana 70803 USA; University of Aberdeen, United Kingdom

**Keywords:** crystal structure, acetamino­phen, acetamino­phen impurity C, cellular oxidants, non-enzymatic biotransformation

## Abstract

In the title compound, C_8_H_8_ClNO_2_, the acetamide substituent is twisted out of the phenyl plane, forming a dihedral angle of 58.61 (7)°. In the extended structure, each mol­ecule donates two hydrogen bonds [N—H⋯O(carbon­yl) and O—H⋯O(carbon­yl)] and thus also accepts two such hydrogen bonds. The chlorine atom is not involved in the hydrogen bonding.

## Abstract 2

In the title compound, the acetamide substituent is twisted out of the phenyl plane, forming a dihedral angle of 58.61 (7)°. In the extended structure, each mol­ecule donates two hydrogen bonds [N—H⋯O(carbon­yl) and O—H⋯O(carbon­yl)] and thus also accepts two such hydrogen bonds. The chlorine atom is not involved in the hydrogen bonding.

## Graphical abstract

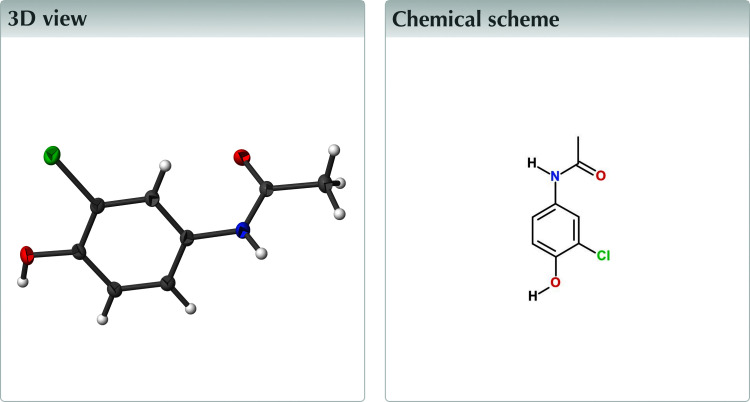

## Structure description

The title compound, C_8_H_8_ClNO_2_, is one of several products formed when acetamino­phen [*N*-(4-hy­droxy­phen­yl)acetamide; C_8_H_9_NO_2_] reacts with hypo­chlorous acid/hypochlorite (HOCl/^−^OCl; pK_a_ ≃7.5) under mildly oxidative, near-neutral pH conditions (Bedner & MacCrehan, 2006 ▸). Ring-chlorination products such as *N*-(3-chloro-4-hy­droxy­phen­yl)acetamide have been detected when wastewater and surface water samples were spiked with environmentally relevant concentrations of acetamino­phen and then subjected to chlorine-based disinfection (Cao *et al.*, 2016 ▸; Kolpin *et al.*, 2002 ▸; Paíga *et al.*, 2025 ▸). Although trichlorinated acetamino­phen does not form under these conditions, chlorination predominantly yields mono- and dichlorinated congeners together with 1,4-benzo­quinone­imine, 1,4-benzo­quinone, and some high-mol­ecular-weight products with *m*/*z* values between 320 and 610 (Bedner & MacCrehan, 2006 ▸; Glassmeyer & Shoemaker, 2005 ▸; Li *et al.*, 2022 ▸). These products possess greater toxicological potency or environmental persistence, prompting researchers to adopt combined and advanced oxidation processes for more efficient removal and reduced toxicity in treated waters (Dahlin & Nelson, 1982 ▸; Postigo & Richardson, 2014 ▸; Qutob *et al.*, 2022 ▸; Vo *et al.*, 2019 ▸; Wang *et al.*, 2020 ▸).

Besides, *N*-(3-chloro-4-hy­droxy­phen­yl)acetamide can appear in acetamino­phen produced *via* the thionyl chloride/SO_2_ Beckmann route, but its formation is effectively suppressed by adding iodide scavengers such as 0.2% KI (Bevan, 1989 ▸) or by using non-chlorinated syntheses followed by double recrystallization of the bulk drug (Abdelmonem *et al.*, 2004 ▸). Because aromatic chloro-acetanilides carry toxicological alerts, pharmacopeias and ICH guidelines limit *N*-(3-chloro-4-hy­droxy­phen­yl)acetamide to < 0.05% and the genotoxic *N*-(4-chloro­phen­yl)acetamide to ≤ 0.001% in finished products (Eur Ph, 2024 ▸; USP, 2024 ▸).

Acetamino­phen is the active ingredient in more than 600 over-the-counter analgesic–anti­pyretic products, with about 25 billion doses sold annually in the United States (Uppu & Fronczek, 2025 ▸; Yoon *et al.*, 2016 ▸). To clarify the mol­ecular structure of its chlorinated impurity, *N*-(3-chloro-4-hy­droxy­phen­yl)acetamide, and to guide studies of its potential biological inter­actions, we grew single crystals of the impurity from water and analyzed them by single-crystal X-ray diffraction.

**Figure 1:**
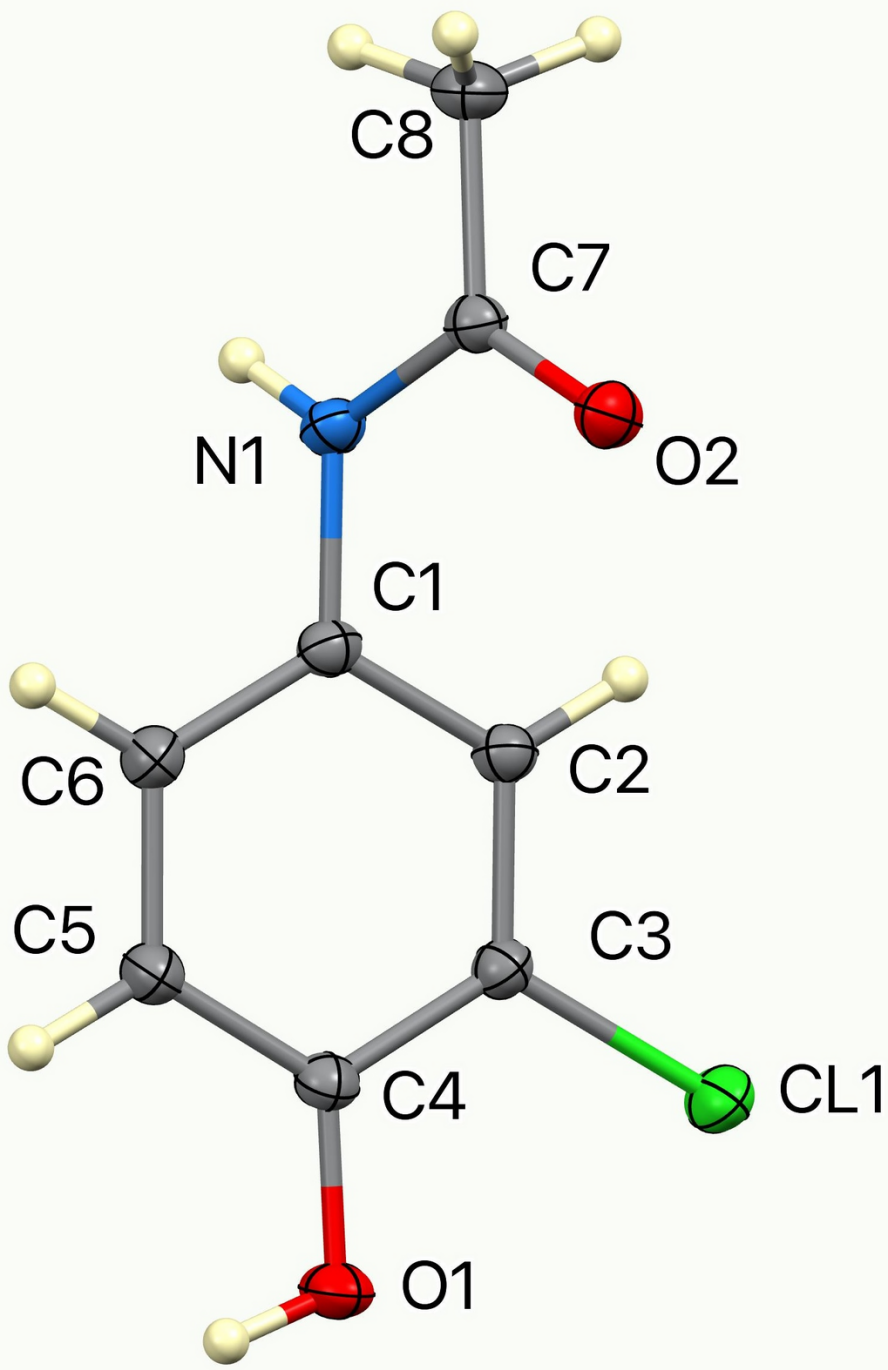
The asymmetric unit of the title compound with 50% displacement ellipsoids.

The mol­ecular structure (Fig. 1 ▸) shows that the hy­droxy oxygen atom O1, the chloro substituent, and the acetamide nitro­gen atom are essentially coplanar with the C1–C6 aromatic ring, having a mean deviation of 0.026 Å. The five-atom acetamide group forms a dihedral angle of 58.61 (7)° with the phenyl group, with the C2—C1—N1—C7 torsion angle being −56.10 (14)° and C1—N1—C7—O1 = −4.77 (15)°.

**Figure 2:**
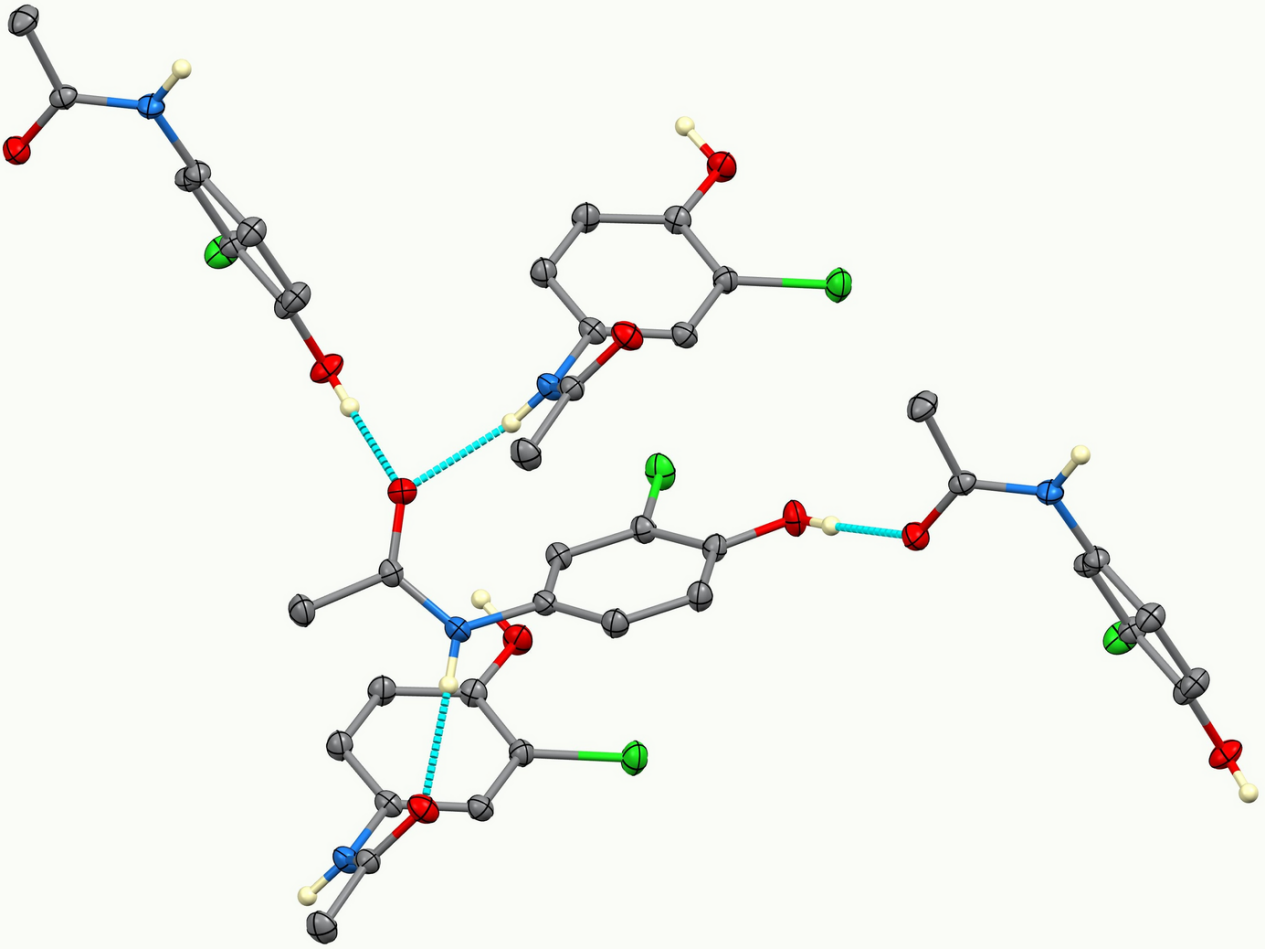
The hydrogen bonding. Only N—H and O—H hydrogen atoms are shown.

**Figure 3:**
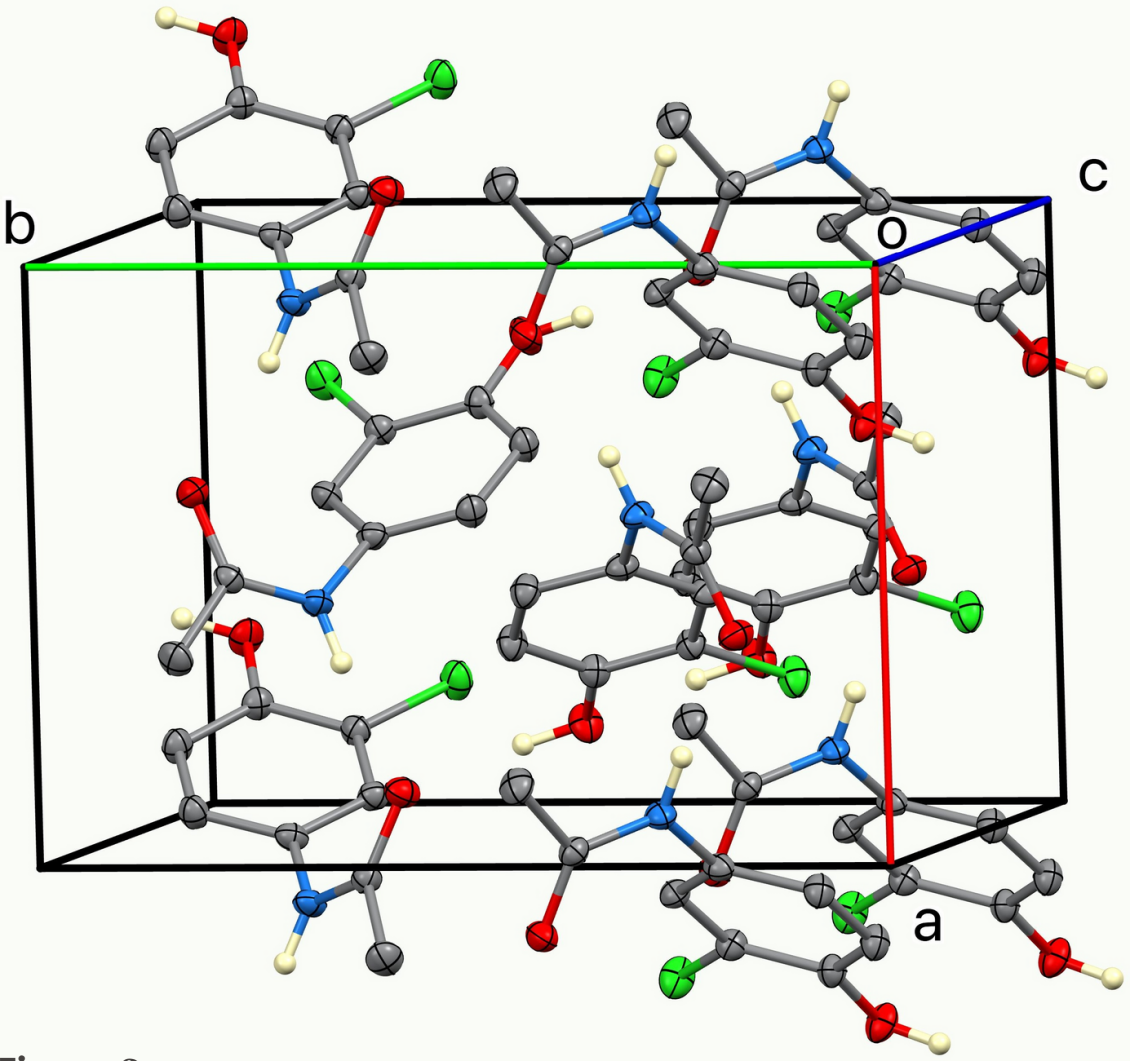
The unit cell. Only N—H and O—H hydrogen atoms are shown.

Inter­molecular inter­actions (Table 1 ▸) are dominated by nearly linear O—H⋯O and N—H⋯O hydrogen bonds from both donors to the acetanilide carbonyl oxygen atom O2. Thus, each mol­ecule donates two hydrogen bonds and accepts two, as shown in Fig. 2 ▸. The O1—H⋯O2(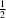 − *x*, *y* − 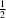, 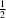 + *z*) hydrogen bond has an O⋯O distance of 2.644 (2) Å and forms chains in the [01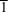] direction with graph-set motif 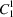
(9). The N1—H⋯O2(*x* + 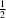, 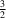 − *y*, *z*) hydrogen bond has an N⋯O distance of 2.866 (2) Å and forms chains in the [100] direction with graph set 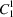
(4). Thus, the overall strong hydrogen bonding pattern is three-dimensional. The chlorine atom is not involved in the hydrogen bonding, as shown in Fig. 3 ▸. A weaker C8—H8*C*⋯O1(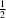 + *x*, 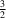 − *y*, 1 − *z*) inter­action exists with C⋯O = 3.250 (2) Å.

## Synthesis and crystallization

*N*-(3-Chloro-4-hy­droxy­phen­yl)]acetamide, C_8_H_8_ClNO_2_ (CAS 3964–54-3) was obtained from AmBeed (Arlington Heights, IL) and was used without further purification. Crystals in the form of yellow needles were prepared by slow cooling of a nearly saturated solution of the title compound in boiling deionized water (resistance *ca*. 18 *M*Ω.cm^−1^).

## Refinement

Crystal data, data collection and structure refinement details are summarized in Table 2 ▸.

## Supplementary Material

Crystal structure: contains datablock(s) I. DOI: 10.1107/S2414314625006959/hb4530sup1.cif

Structure factors: contains datablock(s) I. DOI: 10.1107/S2414314625006959/hb4530Isup2.hkl

Supporting information file. DOI: 10.1107/S2414314625006959/hb4530Isup3.cml

CCDC reference: 2478003

Additional supporting information:  crystallographic information; 3D view; checkCIF report

## Figures and Tables

**Table 1:** Hydrogen-bond geometry (Å, °)

*D*—H⋯*A*	*D*—H	H⋯*A*	*D*⋯*A*	*D*—H⋯*A*
O1—H1*O*⋯O2^i^	0.88 (3)	1.77 (3)	2.644 (2)	172 (4)
N1—H1*N*⋯O2^ii^	0.86 (3)	2.02 (3)	2.866 (2)	168 (3)

Symmetry codes: (i) 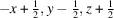; (ii) 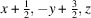.

**Table 2:** Experimental details

Crystal data	
Chemical formula	C_8_H_8_ClNO_2_
*M*_r_	185.60
Crystal system, space group	Orthorhombic, *Pna*2_1_
Temperature (K)	100
*a*, *b*, *c* (Å)	8.0264 (4), 11.6750 (6), 9.1911 (5)
*V* (Å^3^)	861.28 (8)
*Z*	4
Radiation type	Ag *K*α, λ = 0.56086 Å
μ (mm^−1^)	0.21
Crystal size (mm)	0.39 × 0.14 × 0.14

Data collection	
Diffractometer	Bruker D8 Venture DUO with Photon III C14
Absorption correction	Multi-scan (*SADABS*; Krause *et al.*, 2015 ▸)
*T*_min_, *T*_max_	0.841, 0.971
No. of measured, independent and observed [*I* > 2σ(*I*)] reflections	39597, 3610, 3269
*R*_int_	0.141
(sin θ/λ)_max_ (Å^−1^)	0.794

Refinement	
*R*[*F*^2^ > 2σ(*F*^2^)], *wR*(*F*^2^), *S*	0.035, 0.090, 1.05
No. of reflections	3610
No. of parameters	116
No. of restraints	1
H-atom treatment	H atoms treated by a mixture of independent and constrained refinement
Δρ_max_, Δρ_min_ (e Å^−3^)	0.41, −0.20
Absolute structure	Flack *x* determined using 1397 quotients [(*I*^+^)−(*I*^−^)]/[(*I*^+^)+(*I*^−^)] (Parsons *et al.*, 2013 ▸)
Absolute structure parameter	0.13 (6)

Computer programs: *APEX5* and *SAINT* (Bruker, 2016 ▸), *SHELXT2018/2* (Sheldrick, 2015 ▸a), *SHELXL2019/1* (Sheldrick, 2015 ▸b), *Mercury* (Macrae *et al.*, 2020 ▸) and *publCIF* (Westrip, 2010 ▸).

## full crystallographic data

### N-(3-Chloro-4-hydroxyphenyl)acetamide Crystal data

**Table:**

C_8_H_8_ClNO_2_	*D*_x_ = 1.431 Mg m^−^^3^
*M**_r_* = 185.60	Ag *K*α radiation, λ = 0.56086 Å
Orthorhombic, *P**n**a*2_1_	Cell parameters from 4651 reflections
*a* = 8.0264 (4) Å	θ = 3.0–26.3°
*b* = 11.6750 (6) Å	µ = 0.21 mm^−^^1^
*c* = 9.1911 (5) Å	*T* = 100 K
*V* = 861.28 (8) Å^3^	Needle, colourless
*Z* = 4	0.39 × 0.14 × 0.14 mm
*F*(000) = 384	

### N-(3-Chloro-4-hydroxyphenyl)acetamide Data collection

**Table:**

Bruker D8 Venture DUO with Photon III C14 diffractometer	3269 reflections with *I* > 2σ(*I*)
Radiation source: IµS 3.0 microfocus	*R*_int_ = 0.141
φ and ω scans	θ_max_ = 26.4°, θ_min_ = 2.2°
Absorption correction: multi-scan (SADABS; Krause *et al.*, 2015)	*h* = −12→12
*T*_min_ = 0.841, *T*_max_ = 0.971	*k* = −18→18
39597 measured reflections	*l* = −14→14
3610 independent reflections	

### N-(3-Chloro-4-hydroxyphenyl)acetamide Refinement

**Table:**

Refinement on *F*^2^	Hydrogen site location: mixed
Least-squares matrix: full	H atoms treated by a mixture of independent and constrained refinement
*R*[*F*^2^ > 2σ(*F*^2^)] = 0.035	*w* = 1/[σ^2^(*F*_o_^2^) + (0.0412*P*)^2^ + 0.050*P*] where *P* = (*F*_o_^2^ + 2*F*_c_^2^)/3
*wR*(*F*^2^) = 0.090	(Δ/σ)_max_ < 0.001
*S* = 1.05	Δρ_max_ = 0.41 e Å^−^^3^
3610 reflections	Δρ_min_ = −0.20 e Å^−^^3^
116 parameters	Absolute structure: Flack *x* determined using 1397 quotients [(*I*^+^)-(*I*^-^)]/[(*I*^+^)+(*I*^-^)] (Parsons *et al.*, 2013)
1 restraint	Absolute structure parameter: 0.13 (6)

### N-(3-Chloro-4-hydroxyphenyl)acetamide Special details

**Table:**

Geometry. All esds (except the esd in the dihedral angle between two l.s. planes) are estimated using the full covariance matrix. The cell esds are taken into account individually in the estimation of esds in distances, angles and torsion angles; correlations between esds in cell parameters are only used when they are defined by crystal symmetry. An approximate (isotropic) treatment of cell esds is used for estimating esds involving l.s. planes.
Refinement. All H atoms were located in difference maps and those on C were thereafter treated as riding in geometrically idealized positions with C—H distances of 0.95 Å for phenyl and 0.98 Å for methyl. The coordinates of the N—H and O—H hydrogen atoms were refined. *U*_iso_(H) values were assigned as 1.2*U*_eq_ for the attached atom (1.5 for methyl and OH). A torsional parameter was refined for the methyl group. The absolute structure (Parsons *et al.*, 2013) was determined using 1397 quotients, *x* = 0.13 (6).

### N-(3-Chloro-4-hydroxyphenyl)acetamide Fractional atomic coordinates and isotropic or equivalent isotropic displacement parameters (Å^2^)

**Table:**

	*x*	*y*	*z*	*U*_iso_*/*U*_eq_	
Cl1	0.27874 (6)	0.82350 (4)	0.83454 (6)	0.02458 (11)	
O1	0.20435 (18)	0.58074 (12)	0.81799 (15)	0.0226 (3)	
H1O	0.171 (4)	0.510 (3)	0.800 (4)	0.034*	
O2	0.40669 (17)	0.86881 (12)	0.28796 (16)	0.0216 (2)	
N1	0.59913 (19)	0.74091 (13)	0.36310 (16)	0.0180 (3)	
H1N	0.697 (4)	0.712 (2)	0.352 (4)	0.022*	
C1	0.5004 (2)	0.69771 (15)	0.47984 (18)	0.0169 (3)	
C2	0.4449 (2)	0.77256 (15)	0.58827 (19)	0.0174 (3)	
H2	0.473800	0.851413	0.584791	0.021*	
C3	0.3474 (2)	0.73088 (15)	0.70076 (17)	0.0171 (3)	
C4	0.3030 (2)	0.61509 (15)	0.70743 (17)	0.0176 (3)	
C5	0.3617 (2)	0.54145 (15)	0.5997 (2)	0.0200 (3)	
H5	0.334517	0.462332	0.604003	0.024*	
C6	0.4598 (2)	0.58210 (15)	0.48598 (19)	0.0195 (3)	
H6	0.498652	0.531098	0.412947	0.023*	
C7	0.5491 (2)	0.82626 (14)	0.27705 (18)	0.0166 (3)	
C8	0.6697 (2)	0.86814 (17)	0.1643 (2)	0.0235 (3)	
H8A	0.771327	0.821706	0.167850	0.035*	
H8B	0.697519	0.948435	0.183612	0.035*	
H8C	0.618996	0.861631	0.067608	0.035*	

### N-(3-Chloro-4-hydroxyphenyl)acetamide Atomic displacement parameters (Å^2^)

**Table:**

	*U*^11^	*U*^22^	*U*^33^	*U*^12^	*U*^13^	*U*^23^
Cl1	0.0317 (2)	0.02167 (17)	0.02040 (16)	−0.00217 (15)	0.00738 (17)	−0.00433 (16)
O1	0.0280 (6)	0.0215 (5)	0.0184 (6)	−0.0049 (5)	0.0069 (5)	0.0013 (5)
O2	0.0180 (5)	0.0217 (6)	0.0250 (6)	0.0028 (5)	0.0016 (5)	0.0043 (5)
N1	0.0144 (6)	0.0198 (6)	0.0197 (6)	0.0009 (5)	0.0036 (4)	0.0022 (5)
C1	0.0145 (6)	0.0198 (7)	0.0163 (6)	0.0012 (6)	0.0016 (5)	0.0021 (5)
C2	0.0161 (6)	0.0183 (7)	0.0177 (6)	−0.0010 (5)	0.0019 (5)	0.0009 (5)
C3	0.0183 (7)	0.0175 (6)	0.0155 (6)	−0.0002 (5)	0.0019 (5)	−0.0013 (5)
C4	0.0199 (7)	0.0176 (6)	0.0152 (6)	−0.0005 (6)	0.0010 (5)	0.0023 (5)
C5	0.0241 (7)	0.0166 (6)	0.0194 (7)	−0.0010 (6)	0.0036 (6)	0.0013 (5)
C6	0.0221 (8)	0.0175 (7)	0.0190 (6)	0.0007 (6)	0.0037 (6)	−0.0004 (5)
C7	0.0170 (7)	0.0172 (6)	0.0156 (6)	−0.0013 (5)	0.0015 (5)	−0.0005 (5)
C8	0.0248 (8)	0.0238 (8)	0.0220 (7)	−0.0008 (7)	0.0071 (6)	0.0040 (6)

### N-(3-Chloro-4-hydroxyphenyl)acetamide Geometric parameters (Å, º)

**Table:**

Cl1—C3	1.7276 (17)	C2—H2	0.9500
O1—C4	1.349 (2)	C3—C4	1.399 (2)
O1—H1O	0.88 (3)	C4—C5	1.393 (2)
O2—C7	1.251 (2)	C5—C6	1.392 (2)
N1—C7	1.334 (2)	C5—H5	0.9500
N1—C1	1.426 (2)	C6—H6	0.9500
N1—H1N	0.86 (3)	C7—C8	1.500 (3)
C1—C6	1.390 (2)	C8—H8A	0.9800
C1—C2	1.398 (2)	C8—H8B	0.9800
C2—C3	1.385 (2)	C8—H8C	0.9800
			
C4—O1—H1O	108 (2)	C6—C5—C4	120.97 (16)
C7—N1—C1	122.91 (15)	C6—C5—H5	119.5
C7—N1—H1N	120 (2)	C4—C5—H5	119.5
C1—N1—H1N	117 (2)	C1—C6—C5	119.63 (16)
C6—C1—C2	120.20 (16)	C1—C6—H6	120.2
C6—C1—N1	120.31 (16)	C5—C6—H6	120.2
C2—C1—N1	119.49 (16)	O2—C7—N1	121.61 (16)
C3—C2—C1	119.51 (16)	O2—C7—C8	121.04 (16)
C3—C2—H2	120.2	N1—C7—C8	117.34 (15)
C1—C2—H2	120.2	C7—C8—H8A	109.5
C2—C3—C4	121.07 (15)	C7—C8—H8B	109.5
C2—C3—Cl1	119.45 (13)	H8A—C8—H8B	109.5
C4—C3—Cl1	119.49 (13)	C7—C8—H8C	109.5
O1—C4—C5	123.39 (16)	H8A—C8—H8C	109.5
O1—C4—C3	118.01 (15)	H8B—C8—H8C	109.5
C5—C4—C3	118.60 (15)		
			
Cl1—C3—C2—C1	−179.95 (9)	C1—C2—C3—C4	−0.25 (14)
Cl1—C3—C4—O1	1.31 (12)	C1—C6—C5—C4	0.30 (16)
Cl1—C3—C4—C5	−179.02 (10)	C2—C1—N1—C7	−56.10 (14)
O1—C4—C3—C2	−178.39 (11)	C2—C1—C6—C5	0.77 (15)
O1—C4—C5—C6	178.35 (13)	C2—C3—C4—C5	1.29 (15)
O2—C7—N1—C1	−4.77 (15)	C3—C2—C1—C6	−0.79 (15)
N1—C1—C2—C3	179.25 (11)	C3—C4—C5—C6	−1.31 (15)
N1—C1—C6—C5	−179.27 (12)	C6—C1—N1—C7	123.94 (13)
C1—N1—C7—C8	176.31 (12)		

### N-(3-Chloro-4-hydroxyphenyl)acetamide Hydrogen-bond geometry (Å, º)

**Table:**

*D*—H···*A*	*D*—H	H···*A*	*D*···*A*	*D*—H···*A*
O1—H1*O*···O2^i^	0.88 (3)	1.77 (3)	2.644 (2)	172 (4)
N1—H1*N*···O2^ii^	0.86 (3)	2.02 (3)	2.866 (2)	168 (3)

Symmetry codes: (i) −*x*+1/2, *y*−1/2, *z*+1/2; (ii) *x*+1/2, −*y*+3/2, *z*.
